# The use of telemedicine in palliative care in low- and middle-income countries: A scoping review

**DOI:** 10.1177/02692163261441432

**Published:** 2026-04-26

**Authors:** William Goodman, Mandeep Bhattarai, Hasan Shahzad, Sijia Chen, Shaunna Burke, Matthew Allsop

**Affiliations:** 1 School of Medicine, University of Leeds, UK; 2 Kirkburton Health Centre, Huddersfield, UK; 3 School of Biomedical Sciences, University of Leeds, UK

**Keywords:** palliative care, developing countries, telemedicine, scoping review

## Abstract

**Background:**

Telemedicine may improve access and delivery of palliative care in low and middle-income countries (LMICs). Despite increasing adoption and diverse applications, there is limited understanding of their use in palliative care.

**Aim:**

Determine the challenges, enablers and key characteristics of telemedicine interventions targeting outcomes delivered in palliative care in LMICs.

**Design:**

A scoping review guided by the JBI methodology.

**Data sources:**

Six databases (MEDLINE, EMBASE, PsycInfo, Global Health, CINAHL and IEEE Xplore) were searched for reports published between 2003 and August 2025. WHO IRIS and clinical trial registries were searched in March 2026. Forward and backward citation searching was also conducted.

**Results:**

Of the 7938 reports identified from database searches, 21 met the inclusion criteria. Additional searches of WHO IRIS and clinical trial registries did not identify any additional reports. Most interventions focused on telemonitoring (*n* = 14), commonly delivered via telephone (*n* = 8) and involving a doctor/clinician (*n* = 8). Four studies measured only objective levels of engagement, one study assessed only subjective engagement, while three studies measured both objective and subjective engagement, but the measures used across these studies were heterogeneous. Three studies identified challenges to engaging with telemedicine interventions, including poor digital literacy, lack of a physical exam, and poor internet connectivity.

**Conclusions:**

Telemedicine demonstrates emerging, context-specific potential to support access to palliative care in LMICs, although the current evidence base is limited and concentrated in middle-income and cancer-focused settings. Future research should adopt theoretically informed, system-integrated approaches with consistent evaluation to ensure equitable and sustainable delivery.

**What is already known about this topic?**

- Telemedicine approaches have been successfully utilised in LMICs for areas such as tuberculosis treatment, drug monitoring and mental health services.
- There has been no synthesis of the literature detailing the challenges and enablers of engagement with telemedicine approaches in palliative care in LMICs, which could guide future implementation efforts.

**What this paper adds?**

- Whilst the challenges identified are consistent with those found in other reviews of telemedicine approaches in LMICs, this paper also highlights palliative care specific challenges, such as fear of infection and being too ill to travel.
- The telemedicine interventions lack a theoretical underpinning.

**Implications for practice, theory or policy**

- There is a need for an implementation strategy which considers the identified challenges highlighted within this review of poor digital literacy, lack of a physical exam, and poor internet connectivity.
- It is essential for researchers to utilise existing theoretical frameworks to standardise both the reporting of interventions and the outcomes chosen to evaluate their effectiveness.

## Background

Palliative care is a comprehensive, multidisciplinary approach that aims to improve the quality of life of individuals with progressive, life-limiting illnesses, while also maintaining their health and dignity.^1^ Palliative care aims to prevent and relieve physical, emotional, social, and spiritual suffering associated with chronic or life-threatening illnesses, and is recognised as a fundamental human right.^2,3^ The World Health Organisation estimates that nearly 57 million people require palliative care each year, with the majority of these in low- and middle-income countries (LMICs).^2^ However, only 68% of 194 countries report funding for palliative care services with coverage only reaching half the population in 40% of 194 countries.^2^ Within low- and middle-income countries, there are reported challenges to accessing palliative care services, including inadequate funding; hospitals lacking requisite facilities, technologies and trained professionals; weak integration into healthcare systems; and a rural-urban divide in palliative care provision.^4–6^

Telemedicine approaches could offer solutions to increase access to and provision of palliative care in low- and middle-income countries (LMICs). Telemedicine is defined by the World Health Organisation as the provision of healthcare at distance, with healthcare providers and patients not required to be in the same location.^7^ This can involve the use of technology to exchange information for the diagnosis and treatment of illnesses, as well as the prevention of diseases.^7^ Therefore, telemedicine could be used by LMICs to provide comprehensive and timely access to palliative care in remote areas that do not have access to specialist care.^8^ Telemedicine for follow-up surgical care and chronic disease self-management has been found to be beneficial in LMICs by providing education and support to not only patients and their families but also training for local healthcare workers who may not have the expertise to manage certain conditions.^9,10^ Furthermore, it has facilitated remote symptom monitoring, which can help to reduce loss to follow-up from geographically remote patients.^9^ Telemedicine has been found to be beneficial in LMICs for tuberculosis treatment and drug monitoring, telerehabilitation, and there is emerging research to suggest that it could be beneficial in the provision of mental health services.^11–13^ However, there is less of an understanding of how telemedicine approaches have been used in palliative care, although this has been found to be beneficial in high-income countries.^14^

## Objectives

The aims of this scoping review are to: (1) determine the challenges and enablers associated with the use of telemedicine in the delivery of palliative care in low- and middle-income countries and (2) identify elements of telemedicine approaches targeting patient outcomes in low and middle-income countries.

## Methods

This scoping review adhered to the guidance outlined by the JBI (formerly Joanna Briggs Institute).^15^ The Preferred Reporting Items for Systematic Review and Meta-analysis Protocol Extension for Scoping Reviews (PRISMA-ScR) was used to guide the write-up.^16^ This scoping review was registered with the OSF (https://osf.io/dp6k2).

### Eligibility criteria

To identify relevant papers the eligibility criteria included studies that are:

- Focused on palliative care
- Conducted in low- or middle-income countries
- Detailing the use of telemedicine technology as per the definition
- Written in English
- Full-text reports
- Reported as published from 2003 onwards
- Original papers not including systematic reviews

Palliative care was defined as specialist or generalist care explicitly described by the authors as “palliative care,” “hospice,” “end-of-life care,” “supportive care” delivered with palliative intent, or care targeting relief of suffering and quality of life for people with progressive, life-limiting illness. Studies solely focused on curative disease management without palliative intent or palliative-care-relevant outcomes were excluded. Where conference abstracts were identified, a search was conducted to identify if a full-text version was available. Studies were only included that were published from 2003 onwards to ensure the relevance of the technologies reported.

### Information sources

The following databases were searched: MEDLINE, EMBASE, PsycInfo, Global Health, CINAHL and IEEE Xplore. A manual forward and backward citation search was also conducted of the identified reports from the database search. Searches of grey literature sources were conducted in March 2026. This included WHO IRIS and clinical trial registries (ClinicalTrials.gov and the WHO International Clinical Trials Registry Platform). Search terms combined concepts relating to telemedicine, palliative care, and low- and middle-income countries. Records were screened against the same eligibility criteria as the database searches.

### Search

The search strategy was developed by the research team with support from an information specialist from the NIHR Research Design Service at the University of Leeds. The search terms fell under three headings of “palliative care,” “low- or middle-income countries” and “telemedicine.” A review of previous search strategies used in similar fields was conducted, and terms not included in our search strategy were added to refine the searches. The search included reports from 2003 to August 2025 when it was conducted. The MEDLINE search is available in Supplemental Material 1.

### Selection of sources of evidence

When all reports had been identified by the search, duplicate results were removed using EndNote reference manager software. Prior to full screening, training was conducted in which a proportion of titles and abstracts were screened by members of the research team (WG, SB, MB, HS, and MA) to ensure the eligibility criteria were fully understood. Following the training phase, the titles and abstracts were then screened independently in duplicate by two reviewers (WG, SB, MB, HS, and MA) against the eligibility criteria. Any disagreements were settled through discussion or by another member of the research team not involved in the initial screening of that particular report. Full-text copies of reports were obtained and screened independently by two reviewers (WG, SB, MB, HS, and MA) against the eligibility criteria. Similarly, any disagreements were first settled through discussion or by the judgement of another member of the research team not involved in the screening of that particular report. Reasons for exclusion of the full-text reports were recorded and reported.

### Data charting process and items

A standardised extraction form was created in which key information was summarised from the identified reports. Data extraction was conducted by one reviewer (WG) and independently checked by a second reviewer (MA and HS). Discrepancies were resolved through discussion. Items that were extracted included: (i) title, authors, country, year of publication; (ii) Country classification per the Organisation for Economic Co-operation and Development (OECD)^17^; (iii) aim, design; (iv) sample size, demographic and clinical characteristics; (v) description of telemedicine components and intervention, and whether this was underpinned by a theoretical framework; (vi) care settings involved in the use of the technology, healthcare professionals involved in the use of the technology; (vii) details of the data recorded by the technology; (viii) outcomes assessed.

### Data synthesis

A descriptive synthesis was conducted to summarise the study characteristics (country, design, population), and the telemedicine approaches and outcomes used in the individual studies. Country income classification was determined using the Organisation for Economic Co-operation and Development (OECD) Development Assistance Committee (DAC) List of Official Development Assistance (ODA) Recipients corresponding to the year of publication for each study.^17^ As DAC classifications are periodically updated, countries were classified according to the list applicable at the time of publication. Although World Bank income group classifications are commonly used in health systems research, we selected the OECD DAC framework due to its relevance in global health and development contexts. We cross-checked classifications against World Bank income group definitions and identified minor discrepancies for two countries (Tanzania and Bangladesh); however, these differences did not materially alter the overall distribution of studies across income categories. To summarise the range of telemedicine approaches, we applied the established taxonomy by Harst et al., which classifies interventions according to application type (telemonitoring, teleconsultation, telediagnosis, tele ambulance/emergency, and digital self-management); personnel involved; target population; setting; technology; and data provision.^18^ As telediagnosis and tele ambulance/emergency applications were not represented in the included studies, interventions were categorised as telemonitoring, teleconsultation, or digital self-management in accordance with this framework. Outcomes extracted that were related to the engagement with the telemedicine aspect of the intervention were separated into objective (amount, frequency, duration, and depth) and subjective (attention, interest, and affect) outcomes as outlined in previous research.^19^ Extracted data were synthesised in tables and figures.

JBI guidance indicates that critical appraisal in scoping reviews is optional and should be aligned with the review objectives. As the aim of this review was to map the extent, characteristics, and nature of the available evidence rather than to evaluate intervention effectiveness, quality appraisal was not conducted.

## Results

**Figure 1.**
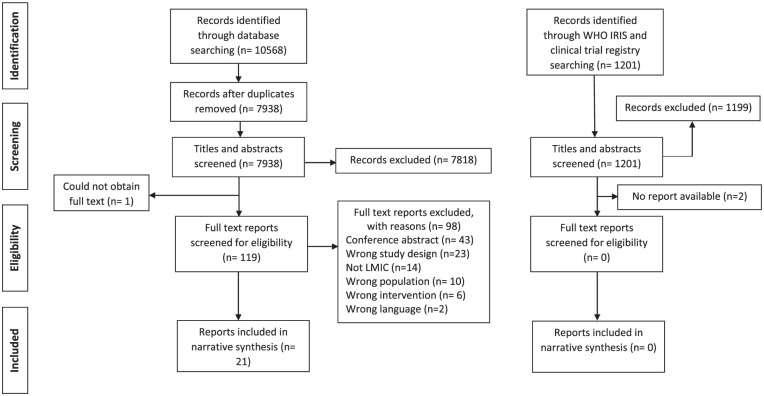
PRISMA flow diagram.

The search strategy identified 10,568 reports from the database search, of which 7938 remained after duplicates were removed. From the search of WHO IRIS and the clinical trial registries 1201 records were identified. After screening was completed 21 reports, covering 20 different studies were eligible for inclusion.^20–40^ The searches of WHO IRIS and trial registries identified additional records; however, none met the inclusion criteria and no reports were included. The breakdown of the screening, along with the reasons for exclusion of the full-text reports, can be found in Figure 1.

### Characteristics of included reports

Eligible reports were conducted across multiple countries, with a plurality in India (*n* = 8)^20–23,26,27,31,36^ and the majority (*n* = 12)^20–23,25–27,31,33,36^ in lower-middle-income countries, while only two^34,35^ were from the least developed countries. Sample size of the included reports ranged from 2 to 1164,^27,35^ with a variety of different study designs used, including: randomised controlled trials (*n* = 7),^28,30,32–34,39,40^ retrospective designs (*n* = 6),^20,24,26,35–37^ prospective studies (*n* = 2),^23,29^ pre-post (*n* = 2),^25,38^ case studies (*n* = 2),^22,27^ a pilot study (*n* = 1)^31^ and a cross-sectional survey (*n* = 1).^21^ The majority of reports focused on patients with advanced cancer (*n* = 14),^20–27,29,31,34,35,37–39^ with others addressing heart failure^28,33^ and end-stage renal disease.^30,32,40^ Further information on the characteristics of the interventions can be found in Table 1.

**Table 1.** Characteristics of included reports (*n* = 21).

First author, year & country	Country classification per the OECD	Study design	Sample size	Participant sociodemographic characteristics	Participant clinical characteristics
Adhikari, 2021; India	Lower-middle income	Retrospective	547	**Age:** Over 18 years old *N* = 532 (97%), under 18 years old *N* = 15 (3%); **Sex:** Male *N* = 263 (48%), Female *N* = 284 (52%)	Various advanced cancer types; **Duration of disease:** Under 6 months *N* = 192 (35%), 6 months to 1 year *N* = 239 (44%), over a year *N* = 54 (21%)
Atreya, 2020; India	Lower-middle income	Cross-sectional survey	50	**Age:** Under 18 years of age *N* = 1 (2%), 19–60 years old *N* = 27 (54%), over 60 years old *N* = 22 (44%); **Sex:** Male *N* = 28 (56%), Female *N* = 22 (44%).	Advanced cancer
Biswas, 2020; India	Lower-middle income	Prospective study and case studies	314	**Age:** Mean 45.4 (SD = 15.6); **Sex:** Male *N* = 148 (47%), Female *N* = 166 (53%)	Various advanced cancer types; **Time since diagnosis:** Under 6 months *N* = 147 (47%), 6 months to 1 year *N* = 101 (32%), over a year *N* = 66 (21%)
Chavarri-Guerra, 2021; Mexico	Upper-middle income	Retrospective	45	**Age:** Median 68 (range 33–90); **Sex:** Male *N* = 19 (42%), Female *N* = 26 (58%).	Various advanced cancer types
Cornetta, 2023; Kenya	Lower-middle income	Pre-post	30	**Age:** 20–39 *N* = 4 (13%), 40–59 *N* = 10 (33%), 60–79 *N* = 10 (33%), 80–93 *N* = (20%); **Gender:** Male *N* = 8 (27%), Female *N* = 22 (73%)	Various advanced cancer types
Dhiliwal, 2022; India	Lower-middle income	Retrospective service evaluation	250	**Age:** Under 20 *N* = 1 (0.4%), 21–40 *N* = 34 (14%), 41–60 *N* = 115 (46%), 61–80 *N* = (92 (37%), over 80 *N* = 8 (3%); **Gender:** Male *N* = 82 (33%), Female *N* = 168 (67%)	Various advanced cancer types
Dhiliwal, 2015; India	Lower-middle income	Case studies	2	60-year-old female and 64-year-old male	Advanced cancer
Domingues, 2011; Brazil	Upper-middle income	Randomised controlled trial	111, IG *N* = 48, CG *N* = 63	**Age:** IG – Mean 62(SD = 12), CG – Mean 63 (SD = 13); **Sex:** IG – Male *N* = 32 (67%), Female *N* = 16 (33%), CG – Male *N* = 32 (51%), Female *N* = 31 (49%), **Ethnicity:** Caucasian IG – *N* = 38 (79%), CG – *N* = 52 (82%)	Heart failure
Hennemann-Krause, 2015; Brazil	Upper-middle income	Prospective study	12	**Age:** Mean 68 years (SD = 9); **Sex:** Male *N* = 7 (58%), Female *N* = 5 (42%)	Various advanced cancer types
Jahromi, 2015; Iran	Upper-middle income	Randomised controlled trial	60, IG *N* = 30, CG *N* = 30	**Age:** Mean 69 (SD = 12); **Sex:** IG – Male (44%), Female (56%), CG – Male (60%), Female (40%)	Advanced chronic renal disease
Kannan, 2013; India	Lower-middle income	Pilot study	60	**Age:** Range 18–76 years; **Gender:** Male *N* = 25 (42%), Female *N* = 35 (58%)	Various advanced cancer types
Karakus, 2024; Turkey	Upper-middle income	Randomised controlled trial	94, IG = 47, CG = 47	**Age:** Mean 61.6 (SD = 12.0), IG Mean 61.8 (SD = 13.5), CG Mean 61.3 (SD = 10.4); **Gender:** Male *N* = 52 (55.3%), Female *N* = 42 (44.7%), IG Male *N* = 24 (51.1%), Female *N* = 23 (48.9%), CG Male *N* = 28 (59.6%), Female *N* = 19 (40.4%)	Various advanced cancer types
Li, 2014; China	Upper-middle income	Randomised controlled trial	135, IG *N* = 69, CG *N* = 66	**Age:** IG – Mean 57 (SD = 13), CG – Mean 55 (SD = 12); **Gender:** IG – Male *N* = 42 (61%), Female *N* = 27 (39%), CG – Male *N* = 37 (56%), Female *N* = 29 (44%)	End-stage renal failure; **Years on ambulatory peritoneal dialysis:** IG – Mean 3 (SD = 2), CG – Mean 4 (SD = 2)
Mirshahi, 2024; Iran	Lower-middle income	Pilot randomised controlled trial	50, IG *N* = 25, CG *N* = 25	**Age:** IG – Mean 45 (SD = 12), CG – Mean 50 (SD = 10); **Sex:** IG – Male *N* = 13 (52%), Female *N* = 12 (48%), CG – Male *N* = 17 (68%), Female *N* = 8 (32%)	Heart failure
Ngoma, 2021; Tanzania	Least developed	Randomised controlled trial	98, IG *N* = 49, CG *N* = 49	**Age:** IG – Under 25 years *N* = 1 (2%), 26–35 years *N* = 7 (14%), 36–45 years *N* = 15 (31%), 46–55 years *N* = 16 (33%), 56–65 years *N* = 9 (18%), over 66 years *N* = 1 (2%), CG – Under 25 years *N* = 3 (6%), 26–35 years *N* = 8 (16%), 36–45 years *N* = 9 (18%), 46–55 years *N* = 16 (33%), 56–65 years *N* = 11 (22%), over 66 years *N* = 2 (4%); **Sex:** IG – Male *N* = 9 (18%), Female *N* = 40 (82%), CG –Male *N* = 18 (37%), Female *N* = 31 (63%)	Various cancer types
Shabnam, 2018; Bangladesh	Least developed	Retrospective	1164	**Age:** Median 52 years (range 2–105); **Sex:** Male (41%), Female (59%)	88% had a cancer diagnosis
Sharma, 2020; India	Lower-middle income	Retrospective	NR	NR	NR
Tang, 2024; China	Upper-middle income	Retrospective	431	**Age:** Median 61 years (range 29–87); **Sex:** Male *N* = 261 (61%), Female *N* = 170 (39%)	Advanced pancreatic cancer
Tumeh, 2023; Brazil	Upper-middle income	Pre-post	125	**Age:** Median 46 years (range 39–54); **Gender:** all female; **Ethnicity:** Multiracial *N* = 63 (50%), White *N* = 29 (23%), Black *N* = 23 (18%), Asian *N* = 10 (8%)	Incurable breast, cervix, endometrial or vulva cancer
Zhang, 2024; China	Upper-middle income	Randomised controlled trial	160, IG = 80, CG = 80	**Age:** Mean IG 67.8 (SD = 3.9), CG 67.4 (SD = 3.9); **Gender:** IG Male *N* = 42 (52.5%), Female *N* = 38 (47.5%), CG Male *N* = 35 (43.8%), Female *N* = 45 (56.3%)	End-stage renal failure

OECD: organisation for economic co-operation and development; IG: intervention group; CG: control group; *N*: number of participants; SD: standard deviation.

### Interventions characteristics and outcomes measured

Table 2 presents the characteristics of the included interventions. The majority of the telemedicine interventions (*n* = 14)^20–23,25,28–32,34,35,37,38,40^ involved telemonitoring, with four focused on teleconsultation^24,26,27,36^ and two included digital self-management.^33,39^ Most interventions were delivered by doctors or other clinicians (*n* = 8),^20,22,23,25–27,34,35,37^ with nurses^26,28,30,32,33,39^ or a team of specialists^21,24,29,36,38,40^ involved in six studies each. In terms of the technology used in the interventions, telephones were most common (*n* = 8),^21,25,26,28,30,32,35,39^ with six using a smartphone^22,23,27,31,33,37,40^ in the interventions and a further six using a combination of the telephone, smartphones and web-based interventions.^20,24,29,34,36,38^ There were no solely web-based interventions. Only one report mentioned that the intervention was underpinned by a theoretical framework, using the Individual and Family Self-Management Theory.^39^

**Table 2.** Characteristics and outcomes of the interventions.

First author, year & country	Summary of telemedicine components and intervention	Engagement outcomes (Perski framework: objective/subjective dimension)	Other outcomes measured
Adhikari, 2021; India	**Application type:** Telemonitoring. **Personnel involved:** Palliative medicine physician. **Technology used:** Telephone-based, smartphone-based. **Data provision:** Voice-call (real-time), text-based (real-time), video-based (real-time). **Summary:** to provide follow-up to patients with round the clock service phone service. Practitioner could access medical records to provide advice and guidance to medical centres and other services.	Objective: Frequency, amount and depth. Subjective: Satisfaction with service. When calls made.	Complaints addressed during teleconsultation. Challenges to in-person consultation.
Atreya, 2020; India	**Application type:** Telemonitoring. **Personnel involved:** Palliative care team. **Technology used:** Telephone-based. **Data provision:** Voice-call (real-time). **Summary:** to provide symptom control to patients during COVID-19 pandemic.	Subjective: Satisfaction with telemedicine service.	Not measured.
Biswas, 2020; India	**Application type:** Telemonitoring. **Personnel involved:** Palliative care senior resident. **Technology used:** Smartphone-based. **Data provision:** Voice-call (real-time), text-based (real-time), video-based (real-time). **Summary:** to provide a 24/7 service during the COVID-19 pandemic. They provided advice to patients on managing their symptoms and could provide information on other services and how to procure opioids at other health centres if they could not come to the hospital.	Objective: Amount and depth. Subjective: Satisfaction with telemedicine service.	Reason of call. Symptom assessment. Challenges to in-person consultation. Advice provided by healthcare professional.
Chavarri-Guerra, 2021; Mexico	**Application type:** Teleconsultation. **Personnel involved:** Multidisciplinary patient navigator (PN)-led team (palliative care, physical therapy, geriatrics, psychology/psychiatry, oncology, and nutrition). **Technology used:** Telephone-based, smartphone-based, web-based. **Data provision:** Voice-call (real-time), text-based (real-time), video-based (real-time). **Summary:** (a) care needs assessments administered remotely; (b) the MDT met weekly using Zoom to discuss patient needs and proposed interventions; (c) the PN presented patients with intervention plans remotely; and (d) if the interventions were accepted, team members implemented them remotely using various contact methods (video conferences, phone calls, messaging, etc.).	Objective: Amount and depth.	Type of intervention conducted. Assistance received to initiate the intervention. Assistance provided by intervention. Challenges to initiating the intervention. Challenges during the intervention.
Cornetta, 2023; Kenya	**Application type:** Telemonitoring. **Personnel involved:** Palliative care provider. **Technology used:** Telephone-based. **Data provision:** Voice-call (real-time). **Summary:** participants were assessed for symptoms at the time of enrolment then through 8 weekly phone calls. Participants were provided free medications commonly used, which they were educated on the indications for use and precautions. Medication reviews were performed and recorded weekly during the calls. Remaining doses were recorded at 4 and 8 weeks.	Not measured	Symptom assessment. Medication usage.
Dhiliwal, 2022; India	**Application type:** Teleconsultation. **Personnel involved:** Physician or nurse. **Technology used:** Telephone-based. **Data provision:** Voice-call (real-time). **Summary:** Home care teams visited patients. Based on patients’ symptoms they were triaged to be either low, medium or high need. As symptoms and disease progression changed over time so could the frequency of home visits. Caregivers were taught how to provide nursing care and medications were prescribed as necessary. Patients were given an emergency phone line where they could reach a physician or nurse at any time.	Not measured	Symptom assessment. Quality of life. Satisfaction with care (patient and caregiver).
Dhiliwal, 2015; India	**Application type:** Teleconsultation. **Personnel involved:** Palliative homecare doctor. **Technology used:** Smartphone-based. **Data provision:** Video-based (real-time), video-based (store and forward), image-based (store and forward), text-based (real-time). **Summary:** to help manage symptoms and medication.	Not measured	Not measured.
Domingues, 2011; Brazil	**Application type:** Telemonitoring **Personnel involved:** Nurse. **Technology used:** Telephone-based. **Data provision:** Voice-call (real-time). **Summary:** IG received educational nursing intervention during hospitalisation followed by telephone monitoring after discharge and CG received in-hospital intervention only. One phone call a week in the first month after discharge followed by 2 phone calls a month over the following 2 months.	Not measured	Level of heart failure awareness. Self-care knowledge. Visits to the emergency room. Re-hospitalisations. Deaths.
Hennemann-Krause, 2015; Brazil	**Application type:** Telemonitoring. **Personnel involved:** Multidisciplinary team including: physician, nurse, social worker, psychologist, music therapist. **Technology used:** Web-based, telephone-based. **Data provision:** Video-based (real-time), voice-call (real-time). **Summary:** weekly web conferences with the MDT either through web conferencing software or phone calls if quality dropped. Symptoms were assessed and complaints heard. Monitoring continued until the patient’s death.	Objective: Duration, frequency	Symptom assessment.
Jahromi, 2015; Iran	**Application type:** Telemonitoring. **Personnel involved:** Nurse. **Technology used:** Telephone-based. **Data provision:** Voice-call (real-time). **Summary:** IG received telephone follow-up 30 days after dialysis in addition to conventional treatment. The telephone follow-ups were structured and contain the following key subjects: communication, cognition/development, breathing/circulation, nutrition, elimination, sleep, pain/perception, skin/tissue, sexuality/reproduction, activity and psychosocial/spirituality/culture.	Not measured	Depression, anxiety and stress scores.
Kannan, 2013; India	**Application type:** Telemonitoring. **Personnel involved:** NR. **Technology used:** Smartphone-based. **Data provision:** Text-based (real-time). **Summary:** patients were sent an SMS to respond with 3 symptom survey scores. Scores were monitored and feedback provided to patients based upon them.	Objective: Frequency	Symptom assessment.
Karakus, 2024; Turkey	**Application type:** Digital self-management. **Personnel involved:** Nurse. **Technology used:** Telephone-based. **Data provision:** Voice-call (real-time). **Summary:** 2 sessions conducted initially face-to-face (second and third day on ward) with 2 subsequent telemonitoring sessions (after discharge). First 2 sessions focused on evaluating patient fatigue and explaining fatigue management strategies. The first telemonitoring session involved reviewing goals set in previous face-to-face session and set new goals. The second telemonitoring session reviewed the goals set in the previous session and any obstacles or difficulties encountered.	Not measured	Fatigue, well-being and independence in activities of daily living scores.
Li, 2014; China	**Application type:** Telemonitoring. **Personnel involved:** Nurse. **Technology used:** Telephone-based. **Data provision:** Voice-call (real-time). **Summary:** IG received a comprehensive discharge planning protocol prior to discharge and a standardised 6-week post-discharge nurse-led telephone support intervention. The first call was conducted within 72 h after discharge to assess the patient’s status and to give advice. The content of each telephone call was guided by the protocol and the specific problems identified in the predischarge assessment. Any problems they encountered were discussed and referrals made.	Not measured	Quality of life. Healthcare utilisation. Blood chemistry. Complication control.
Mirshahi, 2024; Iran	**Application type:** Digital self-management. **Personnel involved:** Nurse. **Technology used:** Smartphone-based. **Data provision:** Voice-call (real-time), text-based (real-time). **Summary:** IG webinar was conducted each week for 30–45 min for 6 weeks. Week 1: introduction to palliative care for heart failure. Week 2: positive problem-solving. Weeks 3–5: symptom management, dietary habits, medication adherence, physical and emotional symptom management, smoking cessation, physical activity, and relaxation. Week 6: spirituality and decision-making. After each session, patients could share their thoughts and questions about the topic discussed on the webinar. Patients also joined WhatsApp groups where they received standardised content corresponding to each week’s webinar topic. Scenarios sent to the WhatsApp groups to encourage discussion on them synchronously.	Subjective: Patient acceptability (qualitative).	Quality of life. Anxiety. Depression. Emergency department visits.
Ngoma, 2021; Tanzania	**Application type:** Telemonitoring. **Personnel involved:** Clinician. **Technology used:** Telephone-based, smartphone-based, web-based. **Data provision:** Voice-call (real-time), text-based (real-time). **Summary:** 2 groups one randomised to mPalliative Care Link group and the other to a phone-contact group, where the palliative care outcome scale was completed twice a week for 4 months. In the mPCL group an SMS would trigger patients or caregivers to complete the outcome scale which would be reviewed by clinicians who would make recommendations which could trigger a visit from local healthcare workers to provide care. This is similar to the other group apart from a clinician would call to complete the outcome scale.	Not measured	Death. Palliative care outcome scale (physical and emotional symptoms) and amount of missing data. Medication usage. Quality of care.
Shabnam, 2018; Bangladesh	**Application type:** Telemonitoring. **Personnel involved:** Palliative care physician. **Technology used:** Telephone-based. **Data provision:** Voice-call (real-time). **Summary:** Patients were able to call for advice on a range of matters.	Objective: Frequency	Reason for call. Advice provided by physician.
Sharma, 2020; India	**Application type:** Teleconsultation. **Personnel involved:** Home care team. **Technology used:** Telephone-based, smartphone-based. **Data provision:** Voice-call (real-time), video-based (real-time). **Summary:** All patients were followed up by the home care teams through regular phone/video consultations and could call in for any problems. However, they were visited only if their needs fulfilled certain criteria: new patient with multiple problems, patients in the last days of life, severe pain, required a procedure, needed medicine for symptom control.	Not measured	Reason for home visit.
Tang, 2024; China	**Application type:** Telemonitoring. **Personnel involved:** Doctor. **Technology used:** Smartphone-based. **Data provision**: Text-based (real-time). **Summary:** Patients could add their doctors as friends to We-Chat an instant messaging app in China. They could contact them whenever they had problems, and the doctor would reply within a day.	Not measured	Completion rate of chemotherapy. Occurrence of severe adverse events. Response to treatment. Patient survival.
Tumeh, 2023; Brazil	**Application type:** Telemonitoring. **Personnel involved:** Psychologist and oncology team. **Technology used:** Smartphone-based, web-based. **Data provision:** Voice-call (real-time), video-based (real-time). **Summary:** Patients would record weekly their symptoms on the Comfort programme. Symptom scores of 4 would generate an alert message to the patient. Scores above 5 would require a telemedicine appointment to be scheduled. For emotional symptoms a psychosocial intervention was conducted and physical symptoms the oncology team was contacted.	Objective: Frequency	Quality of life. Symptom assessment.
Zhang, 2024; China	**Application type:** Telemonitoring. **Personnel involved:** Medical staff (not specified). **Technology used:** Smartphone-based. **Data provision:** Text-based (store and forward). **Summary:** Prescriptions could be accessed through a mobile app with a pharmacy and delivered to the patients. Hospital staff and family members could also remotely help patients through the app. Patients could leave messages for medical staff who could respond to any reactions encountered over 6 months.	Not measured	Self-efficacy, medication adherence, quality of life, depression, and dialysis adequacy.

IG: intervention group; CG: control group.

When examined in relation to the clinical population, additional patterns emerge. Telemonitoring interventions in chronic organ failure populations, including heart failure and end-stage renal disease, were commonly structured around nurse-led or clinician-supported follow-up models delivered via telephone, with a focus on post-discharge care, symptom tracking, and medication management.^28,30,32,40^ In contrast, interventions targeting advanced cancer populations more frequently involved multidisciplinary teams or physicians and incorporated teleconsultation models to address complex symptom burden and care planning needs.^24,29,38^ Digital self-management approaches were observed in both cancer and heart failure contexts^33,39^ and were nurse-led interventions emphasising educational support, goal-setting, and patient-led engagement. These patterns suggest that workforce configuration and technology modality were aligned not only with intervention type but also with underlying disease trajectory and intensity of clinical need in palliative care settings.

In regard to the outcomes that were measured, nine recorded outcomes data in relation to the engagement with the telemedicine interventions.^20,21,23,24,29,31,33,35,38^ Objective measures of engagement aligned with the behavioural dimensions outlined by Perski et al. (frequency, amount, depth and duration) were recorded more often (*N* = 7)^20,22–24,29,31,35,38^ than the subjective outcomes (interest, affect, attention; *N* = 4).^20–23,33^ Three reports included both objective and subjective engagement outcomes: therefore, four measured objective engagement only and one measured subjective engagement only.

Engagement measures were heterogeneous across studies and were operationalised in different ways. Objective engagement most commonly reflected frequency of contact (e.g. proportion of completed calls or conferences) or duration of intervention exposure. Subjective engagement was typically assessed through measures of patient acceptability or experiential feedback. No study employed a validated engagement-specific instrument, and few explicitly referenced a theoretical framework when measuring engagement.

Due to the interventions for the studies that measured engagement frequency not having a set level of expected engagement or not providing sufficient detail to calculate actual engagement, we are unable to determine whether there was a high level of engagement with the interventions. Where calculable, reported engagement ranged from 67.2%^38^ to 93%^31^ of scheduled or expected interactions over the intervention.

Other studies reported descriptive usage metrics, such as an average of 6.4 web conferences^29^ per participant over the intervention or an average of 3.6 phone calls^35^ with patients. In one study, 420 patients (76.8%) had a single phone call and 127 (23.2%) had multiple phone calls with a healthcare professional.^20^ These figures represent observed usage rather than predefined engagement thresholds.

Other outcomes that were recorded focused mostly around the subjective symptom assessment of the individual,^23,25,26,29–31,33,38–40^ the patients’ healthcare utilisation,^25,28,32–34,37^ and the reason for engaging with the intervention.^20,23,35,36^

### Challenges and enablers to telemedicine interventions

In this review only three studies recorded challenges and enablers to telemedicine interventions expressed by patients with advanced cancer.^20,23,24^ The level of detail of the reported challenges and enablers is what is provided by the studies. Two studies reported challenges to in-person visits and the need for a telemedicine consultation.^23^ Across the 861 participants in both studies the reasons for this included, the sealing of borders (during COVID-19 pandemic; *N* = 391), the patient having a terminal cancer diagnosis (*N* = 88), fear of going to the hospital due to risk of infection (*N* = 275), a lack of social support to help get to the hospital (*N* = 68), and physical or cognitive impairments (*N* = 39).^20,23^ The other study reported challenges around the initiation and during the telemedicine intervention.^24^ Challenges reported for the initiation of the intervention included, the participant lacking experience with the method of communication (*N* = 46 (28%)), participant unavailable (*N* = 8 (5%)), lack of device for communication (*N* = 4 (2%)), no internet connexion (3 (2%)), and poor call quality (*N* = 1 (1%)).^24^ Whereas during the intervention the challenges that were reported were interruptions (*N* = 24 (15%)), poor internet connexion (*N* = 21 (13%)), poor video or audio quality (*N* = 20 (12%)), privacy concerns (*N* = 10 (6%)), fatigue (*N* = 4 (2%)), lack of a physical exam (*N* = 3 (2%)), and hearing problems (*N* = 3 (2%))^24^

## Discussion

### Main findings/results of the study

This scoping review is the first to identify the breadth of telemedicine approaches used in palliative care in LMICs. This review identified 21 different records covering 20 separate studies with the majority conducted in middle income countries. Only three papers identified in this review investigated the challenges and enablers to telemedicine interventions in LMICs but they identified broadly patient level factors such as their terminal illness, fears of going to the hospital and a lack of support to help them get to the hospital, but also broader structural/societal challenges such as a lack of internet access, no access to a device for communication or poor audio/visual quality. While a range of modalities were used within the interventions, the telephone was used in the majority of the studies although it was often paired with complementary technologies such as smartphones or web-based interventions. The healthcare professionals most commonly supporting the interventions were doctors although many involved wider multidisciplinary teams including nurses.

### What this study adds

The World Health Organisation (WHO) has developed a Global Strategy on Digital Health to enhance the use of digital tools and innovations in health systems, particularly benefitting LMICs to enable them to achieve universal healthcare (including palliative care) through the use of digital health technology.^41^ The strategy emphasises the potential of digital technologies to bridge gaps in healthcare delivery, improve access, and enhance the quality of health services in resource-constrained settings by promoting the integration and interoperability of digital solutions, such as telemedicine, mobile health applications, and data analytics, to strengthen health systems, improve disease surveillance, and support healthcare workers. Despite the push to increase the availability and use of telemedicine approaches in LMICs some challenges have been identified. Recommendations on digital interventions for health system strengthening by the WHO found that challenges covered areas such as the health system, societal infrastructure and patient-level factors.^42^ In this review, healthcare providers reported connectivity issues, inability to charge their phone in some instances and client dissatisfaction with connectivity issues causing them to receive less timely care. Clients also reported not having access to mobile phones, with some gender discrimination reported as a reason for this, but also changing their number and not reporting this to their healthcare provider. Across both healthcare providers and clients, a lack of digital literacy was reported, which reduced the uptake of these services. Despite efforts to extend telemedicine in LMICs, including recommendations for its implementation across sub-Saharan Africa within oncology care,^43^ diverse patient and provider challenges persist. The WHO recommend a broad range of targets to enhance the digital ecosystem in LMICs, including strengthening digital infrastructure, building workforce capacity, and securing sustainable funding.^41^ Aspects such as infrastructure need to be addressed immediately so that the application of telemedicine approaches does not perpetuate the unequal distribution of healthcare services to those with access to these technologies and the infrastructure to support them.^44^ However, in the longer term, there is a need to consider broader factors influencing implementation. In palliative care contexts, these structural barriers may be amplified. Patients with advanced illness often experience fatigue, cognitive impairment, or functional decline, increasing reliance on family caregivers to facilitate digital communication.^2,45^ Telemedicine models in LMICs must therefore account not only for infrastructure constraints but also for caregiver capacity, household resources, and the feasibility of sustained engagement for individuals with high symptom burden.

The challenges identified in this review align with the WHO’s phased telemedicine implementation guidance, which emphasises situational assessment, planning, and monitoring and evaluation.^46^ At the situational assessment stage, barriers such as limited access to devices, poor internet connectivity, low digital literacy, and the absence of social support highlight the need to evaluate both the technological and social infrastructure in which palliative care is delivered. Planning and delivery challenges, including poor call quality, interruptions, privacy concerns, and the absence of a physical examination, underline the importance of developing clear clinical protocols, low-bandwidth or alternative communication options, and safeguards for sensitive conversations, particularly around end-of-life decision-making. In palliative care, this is particularly critical for advance care planning conversations, discussions about goals of care, and end-of-life decision-making, where communication quality, privacy, and cultural sensitivity are essential.^47^ In the monitoring and evaluation phase, the need for continuous feedback is evident, as patients with advanced disease often experience fatigue, symptom burden, or sensory impairments that may compromise engagement, and caregivers play a central role in facilitating effective use of telemedicine. Moving forward, situational assessments in low- and middle-income countries should explicitly consider household and community resources, caregiver capacity, and the affordability of digital solutions. Planning for implementation will benefit from embedding telemedicine within existing palliative care networks, including linkages to medicines delivery, referral pathways, and home-based services to ensure continuity of care. For example, digital symptom reporting systems must be linked to reliable escalation pathways, including access to essential medicines and in-person assessment where required, to prevent avoidable symptom crises. Monitoring and evaluation frameworks should extend beyond uptake to capture patient- and caregiver-reported outcomes such as communication quality, dignity, and symptom management. Taken together, these findings illustrate how aligning palliative care-specific considerations with the WHO framework can convert observed barriers into actionable enablers, supporting the development of person-centred and contextually responsive telemedicine models in LMICs.

Although most included studies focused on advanced cancer populations, several studies examined telemedicine in heart failure and end-stage renal disease, offering transferable lessons for broader palliative populations.^28,30,32,33,40^ These interventions commonly employed structured nurse-led telephone monitoring and post-discharge follow-up models aimed at symptom tracking, medication management, and continuity of care. Chronic organ failure trajectories share important features with advanced cancer, including fluctuating symptom burden, repeated hospitalisation, and high caregiver involvement.^45^ The findings from these studies suggest that telemedicine may be particularly suited to supporting longitudinal symptom monitoring and coordinated care for patients with non-malignant progressive conditions, where access to specialist palliative services is often fragmented. This highlights the potential applicability of telemedicine models beyond oncology and reinforces the need to consider diverse disease trajectories when designing and evaluating digital palliative care interventions in LMICs.

Within the studies identified in the present review only 8 of them were evaluating already established telemedicine systems with the other 12 studies evaluating the feasibility and proof-of-concept of telemedicine approaches in palliative care settings. Therefore, there is a need for further research to explore the implementation of these approaches within the healthcare setting to ensure that they can be integrated into the local healthcare system. There has also been a recent push to integrate artificial intelligence (AI) into telemedicine, however, none of the studies in the present review explored this. AI has the ability to support patient monitoring, diagnosis and support decision-making and healthcare delivery.^48^ Future research should explore how implementing AI into telemedicine in palliative care settings particularly in LMICs could help to relieve pressure on clinicians and provide a low-cost solution to expanding healthcare. In palliative care settings, such applications would need to prioritise symptom triage, caregiver support, and culturally appropriate communication rather than purely diagnostic optimisation. Safeguards would also be required to ensure that automated decision-support tools do not replace relational aspects of care that are central to palliative practice. The use of AI could also be leveraged to adapt interventions to suit the cultural sensitivities of the individuals by providing personalised information and expanding healthcare to communities that may feel underserved.^49,50^

Measuring engagement with telemedicine approaches is essential for determining their effectiveness and long-term success, particularly as these technologies become an integral part of healthcare systems.^19^ However, this review identified that only half of the reports measured any engagement measurements with the telemedicine aspects of the interventions. Amongst other populations, such as those with hypertension, diabetes or mental health issues, greater engagement with digital interventions has been associated better health outcomes.^51,52^ Therefore, understanding the level of engagement with telemedicine approaches is important for their implementation in healthcare systems in LMICs and future research should ensure this is measured. In palliative populations, engagement may be influenced by fluctuating symptom burden, caregiver involvement, and proximity to end of life, suggesting that engagement metrics should be interpreted differently from those used in stable chronic disease populations.

Within the present review only one study was identified as being underpinned by a theoretical framework. Theoretical frameworks for digital health interventions can be used to guide the implementation and evaluation of these complex interventions by encouraging the researcher to consider the various interactions between the patients, healthcare professionals and caregivers; the technologies deployed; and the healthcare systems in which they will operate.^53^ They can also help to standardise the reporting of the interventions, and the outcomes needed to evaluate the effectiveness of them.^53^ In palliative care, such frameworks should explicitly account for caregiver roles, dynamic symptom trajectories, cultural norms around decision-making, and integration with home-based or community services. Therefore, future telemedicine interventions in palliative care in LMICs should be underpinned by a theoretical framework to ensure the development of these interventions is informed by evidence.

### Limitations of the study

However, there are limitations with the current review. Despite the aims of this review to synthesise findings from LMICs, only two studies were conducted in low-income countries which limits the generalisability of these findings to these settings. Alternative classification systems (e.g. World Bank income groups) categorised Tanzania and Bangladesh one income category higher than the system used in this review; however, this would not materially change the overall interpretation of the distribution of evidence. The majority of identified reports were conducted in cancer populations which may limit the applicability of findings to other palliative care groups, as different disease trajectories, treatment pathways, and palliative care engagement may influence the suitability and use of telemedicine interventions. Furthermore, this review was limited to English-language publications, which may have introduced language bias. Relevant telemedicine initiatives may have been reported in local-language journals or national publications, particularly in countries with substantial non-English academic output (e.g. Brazil, China, Mexico, Iran), and may therefore not have been captured. It is also possible that studies from LMIC contexts not represented in this review were published only in non-English outlets, leading to potential underrepresentation of implementation activity in certain regions. Although searches of WHO IRIS and trial registries were conducted, grey literature sources such as ministry or NGO implementation reports may remain underrepresented if not indexed or publicly accessible. The absence of formal quality appraisal within this review limits the ability to assess risk of bias or to draw firm conclusions regarding effectiveness. Included studies varied substantially in design, including feasibility studies, retrospective analyses, and randomised controlled trials, and findings should therefore be interpreted cautiously.

In conclusion, this review highlights the emerging but uneven evidence base for telemedicine in palliative care across LMICs, with most studies focused on middle-income settings and cancer populations, leaving knowledge gaps for low-income countries and other disease groups. The challenges and enablers identified resonate with the WHO’s telemedicine implementation guidance and the Lancet Commission on cancer in sub-Saharan Africa, emphasising that structural, societal, and patient-level barriers must be systematically addressed to avoid exacerbating inequities. Importantly, palliative care brings specific requirements, such as caregiver involvement, sensitivity to symptom burden, and the need for safe, private spaces for end-of-life communication, that should be explicitly integrated into digital health strategies. Future research should move beyond feasibility to rigorously evaluate telemedicine interventions that are embedded in health systems, underpinned by theoretical frameworks, and responsive to cultural and contextual realities. By doing so, telemedicine may have the potential not only to expand access but also to strengthen person-centred, equitable models of palliative care delivery in LMICs.

## Supplemental Material

sj-docx-1-pmj-10.1177_02692163261441432 – Supplemental material for The use of telemedicine in palliative care in low- and middle-income countries: A scoping review Supplemental material, sj-docx-1-pmj-10.1177_02692163261441432 for The use of telemedicine in palliative care in low- and middle-income countries: A scoping review by William Goodman, Mandeep Bhattarai, Hasan Shahzad, Sijia Chen, Shaunna Burke and Matthew Allsop in Palliative Medicine
