# Supplementary material for: The use of telemedicine in palliative care in low- and middle-income countries: A scoping review
Source: Palliat Med. 2026 Apr 26;40(7):987–99. doi: 10.1177/02692163261441432 (PMC13323915; doi:10.1177/02692163261441432)
Supplement: sj-docx-1-pmj-10.1177_02692163261441432 – Supplemental material for The use of telemedicine in palliative care in low- and middle-income countries: A scoping review [file sj-docx-1-pmj-10.1177_02692163261441432.docx]

**Supplementary Material One**

**MEDLINE search strategy**

1

exp palliative nursing/ or exp palliative therapy/ or exp palliative care/ or exp palliative medicine/

2

hospice care/ or hospice/ or hospice nursing/ or hospice patient/

3

terminal care/

4

death/ or dying/

5

palliative*.ti,ab.

6

hospice*.ti,ab.

7

(terminal* adj3 (ill* or disease* or patient* or stage* or cancer* or carcinoma* or metasta* or neoplas* or tumor* or tumour* or malignan*)).tw.

8

("end of life" or "end stage").ti,ab.

9

(dying adj3 (care or comfort or relief or strateg* or plan or intervention* or pain?)).ti,ab.

10

(death adj3 (near or impending)).tw.

11

(advanced adj5 (ill* or disease* or patient* or stage* or cancer* or carcinoma* or metasta* or neoplas* or tumor* or tumour* or malignan*)).tw.

12

1 or 2 or 3 or 4 or 5 or 6 or 7 or 8 or 9 or 10 or 11

13

Developing Countries/

14

(low* income* adj3 (countr* or nation* or economy or economies)).tw,kf.

15

(low-resource adj3 setting*).ti,ab.

16

(middle income* adj3 (countr* or nation* or economy or economies)).tw,kf.

17

(low* middle adj3 (countr* or nation* or economy or economies)).tw,kf.

18

(LMIC or LMICs).tw,kf.

19

((LIC or LICs) adj3 (countr* or nation* or economy or economies)).tw,kf.

20

"transition* countr*".tw,kf.

21

((underserved or "under served" or deprived or poor*) adj3 (country or countries or nation? or economy or economies)).tw,kf.

22

((Developing or "under developed" or underdeveloped or "less* developed" or "third world") adj3 (country or countries or nation? or economy or economies)).tw,kf.

23

((Developing or "under developed" or underdeveloped or "less* developed") adj2 world).tw,kf.

24

((Africa* not "African American*") or (Asia* not "Asian American*")).ti,ab,in,kf.

25

(Afghanistan* or Albania* or Algeria* or Angola* or Antigua or Barbuda or Argentin* or Armenia* or Azerbaijan* or Bangladesh* or Belarus* or Belize* or Benin* or Bhutan* or Bolivia* or Bosnia* or Herzegovina or Borneo or Botswana* or Brazil* or "Burkina Faso" or Burundi*).ti,ab,in,kf.

26

(Cambodia* or Cameroon* or "Cape Verde*" or "Cabo Verde*" or Caribbean* or Chad or Chile* or China or Chinese or (Colombia* not "British Colombia*") or Comoros or Congo or "Cook Island*" or "Costa Rica*" or "ivory coast" or "cote d'ivoire" or Cuba* or Djibouti* or Dominica*).ti,ab,in,kf.

27

(Ecuador* or Egypt* or "El Salvador" or Eritrea* or Eswatini or Ethiopia* or Fiji* or Gabon* or Gambia* or Ghana* or Grenada* or Guatemala* or Guinea* or Guyana* or Haiti* or Hondura* or India or (Indian? not "American Indian?") or Indonesia* or Iran* or Iraq*).ti,ab,in,kf.

28

(Jamaica* or Jordan* or Kazakhstan* or Kenya* or Kiribati* or Korea* or DPRK or Kosovo* or Kyrgyz* or "Lao PDR" or "Lao People*" or Laos or Laotian or Lebanon or Lebanese or Lesotho or Liberia* or Libya*).ti,ab,in,kf.

29

(Macedonia* or Madagasca* or Malawi* or Malaysia* or Maldives or Mali or "Marshall Islands" or Mauritania* or Mauritius or Mayotte* or Melanesia* or Mexico or Mexican? or Micronesia* or Moldova* or Mongolia* or Montenegro* or Morocco or Moroccan? or Mozambique* or Myanmar*).ti,ab,in,kf.

30

(Namibia* or Nauru* or Niue* or Nepal* or Nicaragua* or Niger or Nigeria* or Pakistan* or Palau* or Palestin* or Panama or "Papua New Guinea*" or Paraguay or Peru* or Peruvian* or Philippines* or Pilipin* or Filipin* or Rwanda*).ti,ab,in,kf.

31

(Samoa* or "Sao Toms*" or Principe* or Senegal* or Serbia* or Seychelles or "Sierra Leone" or "Solomon Islands" or Somalia* or "Sri Lanka*" or "S* Lucia" or "S* Helena" or "S* Vincent and the Grenadines" or "South America*" or Sudan* or Suriname* or Swaziland* or Syria*).ti,ab,in,kf.

32

(Tajikistan* or Tanzania* or Thai* or Timor* or Togo or Tokelau or Tonga or Tunisia* or Turkey or Turkish or Turkmenistan* or Tuvalu* or Uganda* or Ukrain* or Uruguay* or Uzbekistan* or Vanuatu* or Venezuela* or Vietnam* or "Wallis and Futuna" or Futuna or "West Bank" or Gaza or Yemen* or Zambia* or Zimbabw*).ti,ab,in,kf.

33

exp Africa/

34

caribbean region/ or west indies/ or "antigua and barbuda"/ or cuba/ or dominica/ or dominican republic/ or grenada/ or haiti/ or jamaica/ or saint lucia/ or "saint vincent and the grenadines"/ or central america/ or costa rica/ or el salvador/ or guatemala/ or honduras/ or nicaragua/ or exp panama/ or mexico/ or exp south america/

35

asia/ or exp asia, central/ or asia, southeastern/ or borneo/ or cambodia/ or timor-leste/ or indonesia/ or laos/ or malaysia/ or mekong valley/ or myanmar/ or philippines/ or thailand/ or vietnam/ or asia, western/ or bhutan/ or exp india/ or middle east/ or afghanistan/ or iran/ or iraq/ or jordan/ or lebanon/ or syria/ or turkey/ or yemen/ or nepal/ or pakistan/ or sri lanka/ or far east/ or exp china/ or exp korea/

36

pacific islands/ or melanesia/ or fiji/ or papua new guinea/ or vanuatu/ or micronesia/ or palau/ or polynesia/ or exp samoa/ or tonga/ or philippines/

37

Georgia.ti,ab. not Georgia/

38

(Montserrat not (Spain or Espana)).ti,ab.

39

13 or 14 or 15 or 16 or 17 or 18 or 19 or 20 or 21 or 22 or 23 or 24 or 25 or 26 or 27 or 28 or 29 or 30 or 31 or 32 or 33 or 34 or 35 or 36 or 37 or 38

40

exp Telecommunications/

41

(ehealth* or e-health* or "electronic health*" or mhealth* or m-health* or "mobile health*").tw,kw.

42

(remote* adj3 (monitor* or consultation* or communicat*)).tw,kw.

43

("smart phone*" or smartphone* or iphone* or "cell* phone*" or cellphone* or "mobile phone*" or ipad* or app? or mobile application?).tw,kw.

44

(PDA or handheld or hand-held or "instant messag*" or "text-messag*" or "electronic messag*" or "short messag* service" or phone messag* or text* or SMS or "web 2.0" or "health 2.0" or "medicine 2.0" or email*).tw,kw.

45

exp microcomputers/

46

mobile applications/ or user-computer interface/ or web browser/

47

(Skype or facetime or google or facebook or face-book or blog* or vlog* or videoblog* or "video-blog*" or YouTube or you tube).tw,kw.

48

(webcast* or web-cast* or podcast* or pod-cast* or videocast or video-cast or instagram or whatsapp or snapchat or WeChat or twitter or tweet*).tw,kw.

49

(social media or social network* or social software or chat room* or chatroom* or crowdsourc* or crowd sourc* or cyworld).tw,kw.

50

((electronic* or online or "on-line" or web* or internet or digital*) adj8 (self-report* or patient-report*)).tw,kw.

51

exp internet/ or Crowdsourcing/

52

(bebo or dailystrength or doximity or dropbox or flickr or friendster or gaming or hi5 or hyves or igoogle or instagram or linkedin or myspace or myfamilyhealth or netvibes or orkut or pageflakes or patientslikeme).tw,kw.

53

(picasa or pinterest or plaxo or reddit or renren or researchgate or rss or sciencestage or screencast or slideshare or studiviz or tumblr or vimeo or vodcast or widget or wiki or wordpress).tw,kw.

54

((online or "on-line" or web or website* or web-site* or web-based or internet or digital* or electronic* or video* or telephone*) adj4 (technolog* or intervention* or peer* or support* or group* or program* or monitor* or educat* or communicat* or resource* or tool*)).tw,kw.

55

(telemedicine or tele-medicine or telehealth or tele-health or teleoncology or tele-oncology or telemonitor* or tele-monitor* or telecare or tele-care or telerehabilitation or tele-rehabilitation).tw,kw.

56

(wear* adj5 (monitor* or sensor* or device*)).tw,kw.

57

40 or 41 or 42 or 43 or 44 or 45 or 46 or 47 or 48 or 49 or 50 or 51 or 52 or 53 or 54 or 55 or 56

58

12 and 39 and 57

59

exp Animals/ not exp Humans/

60

exp veterinary medicine/

61

exp animal experimentation/

62

59 or 60 or 61

63

58 not 62
